# Impact of obesity on survival outcomes of women with advanced epithelial ovarian cancer in Lagos, Nigeria: a retrospective cohort study

**DOI:** 10.3332/ecancer.2024.1743

**Published:** 2024-08-20

**Authors:** Kehinde S Okunade, Olukayode O Akinmola, Temitope V Adekanye, Akhenamen Packson, Hameed Adelabu, Olufemi Thomas-Ogodo, Austin C Okoro, Chinelo Okoye, Rose I Anorlu

**Affiliations:** 1 Department of Obstetrics and Gynaecology, College of Medicine, University of Lagos, PMB 12003, Surulere, Lagos, Nigeria; 2 Department of Obstetrics and Gynaecology, Lagos University Teaching Hospital, PMB 12003, Surulere, Lagos, Nigeria; 3 Center for Clinical Trials, Research and Implementation Science, College of Medicine, University of Lagos, PMB 12003, Surulere, Lagos, Nigeria; 4 Department of Clinical Pathology, Lagos University Teaching Hospital, PMB 12003, Surulere, Lagos Nigeria

**Keywords:** advanced EOC, body mass index, overweight, surgical debulking surgery, survival

## Abstract

**Background:**

Epithelial ovarian cancer (EOC) is a major contributor to cancer-related illness and death among women worldwide. Obesity, a prevalent condition in many populations, has been implicated as a risk factor for various malignancies including EOC.

**Objectives:**

This study investigated the impact of obesity on survival outcomes among women with advanced EOC in Lagos, Nigeria.

**Methods:**

We conducted a retrospective analysis of patient medical records from a major gynaecological cancer unit of a teaching hospital in Lagos, Southwest Nigeria, to examine the relationship between body mass index (BMI) 30 kg/m^2^ as a measure of obesity, and progression-free (PFS) and overall survival (OS). We used Kaplan-Meier analysis stratified by patients’ BMI categories (obese versus non-obese) and compared using the Log Rank test to estimate PFS and OS. The multivariable Cox proportional hazard model was used to estimate hazard ratios (HR) of the associations between the BMI categories and survival outcomes while adjusting for all confounding clinicopathologic variables. Hypothesis tests were conducted using a two-tailed approach with a significance level of 5%.

**Results:**

Our study showed no statistically significant association between obesity and PFS (adjusted HR = 0.62, 95% confidence interval = 0.36–1.06, *p* = 0.282). However, a significant association was observed between obesity with or without ascites and OS (adjusted HR = 3.58, 95% confidence interval 1.28–10.02, *p* = 0.015).

**Conclusion:**

Our findings suggest that obesity negatively impacts OS in patients with EOC, thus highlighting the need to address obesity in the management of EOC by introducing comprehensive, multidisciplinary approaches incorporating weight management and personalized treatment strategies to enhance the prognosis of these patients.

## Introduction

Epithelial ovarian cancer (EOC) accounts for the largest histological type of ovarian cancer [1] and is also the most common cause of gynaecological cancer death [1, 2]. It is often diagnosed at an advanced stage due to non-specific symptoms and a lack of effective early screening [3, 4]. Globally, EOC presents a substantial health burden [5, 6], with significant variations in incidence and outcomes based on geographical regions and population demographics [5, 6].

Despite advancements in surgical and chemotherapeutic interventions, the prognosis for women with advanced EOC remains poor, with 5-year survival rates lingering around 20%–40% worldwide [7–9]. Understanding and addressing modifiable risk factors of survival outcomes is crucial for improving prognosis and guiding treatment strategies. These risk factors include obesity, which is defined as a body mass index (BMI) of 30 kg/m² or higher [10]. It has emerged as a significant public health concern [10–12] and a potential risk factor for various cancers [13–17], including EOC [18]. The global prevalence of obesity has more than doubled since the 1980s [10, 12, 19], and it continues to rise, with significant variations across different regions [10, 12, 20]. The link between obesity and cancer outcomes is complex and multi-dimensional, encompassing hormonal imbalances, chronic inflammation, changes in adipokine levels and metabolic dysregulation [17, 21]. These factors may contribute to more aggressive tumour biology and poorer response to treatment in obese patients.

Obesity is also rising in Nigeria [22, 23], reflecting broader global trends and posing additional challenges to public health systems [11, 19, 24]. This is partly due to the high prevalence of sedentary lifestyles and the increase in processed food outlets, which is a trend observed in many African settings [23]. However, the impact of obesity on survival outcomes specifically among women with advanced EOC remains underexplored, particularly in low- and middle-income countries such as Nigeria. This study, therefore, assessed the effects of obesity on survival outcomes among women with advanced EOC managed over 10 years at a university teaching hospital in Lagos, the largest city in Nigeria, with its diverse population and healthcare infrastructure, thus providing a unique setting to study the interplay between obesity and cancer outcomes.

## Patients and methods

### Study design and setting

This retrospective cohort study was conducted at the gynaecological oncology unit of the Lagos University Teaching Hospital (LUTH) to extract and analyse the data of women with advanced EOC managed from January 2008 to December 2017. LUTH is the leading tertiary healthcare facility in Lagos, Southwest Nigeria. It primarily serves as a specialized referral centre for both public and private hospitals in Lagos and the neighbouring Ogun and Oyo States. The hospital's gynaecological oncology unit has four consultant gynaecological oncologists and up to 15 resident doctors. They offer a range of multidisciplinary oncology services for both in-patient and out-patient care, including the diagnosis and treatment of various premalignant and malignant diseases of the female genital tract [25, 26].

### Eligibility criteria

We retrieved data from the medical records of women diagnosed and treated for EOC in the oncology unit during the review period. We additionally collected data on tumour recurrence and mortality for up to 3 years following the completion of treatment, until December 2020. Included patients were those with surgical-pathological evidence of advanced EOC (FIGO – International Federation of Gynaecology and Obstetrics stages III and IV) [27]. Also, those who had complete primary treatment comprising either preoperative neoadjuvant adjuvant chemotherapy, interval debulking surgery (IDS) and postoperative adjuvant chemotherapy (ACT) or primary debulking surgery (PDS) and postoperative (ACT); and those with complete clinical data required in the final data analyses. Women who did not have their mass index (BMI) recorded at diagnosis; those without sufficient evidence of treatment completion; and those who were lost to follow-up were excluded from the final analysis.

### Study procedure and data collection

We obtained complete data for 126 women diagnosed with advanced EOC from the gynaecologic oncology ward register and their medical records. Extracted data included the patient’s age, menstrual status, parity, BMI, medical comorbidity (including hypertension, diabetes mellitus, kidney and liver disease), presence of significant ascites (up to one litre), preoperative serum CA-125 levels, type of primary surgery (PDS or IDS), surgical debulking status (optimal or suboptimal), presence of significant ascites, FIGO stage (stage III or IV), tumour histological type (Type I – endometrioid carcinoma, clear cell carcinoma, mucinous carcinoma and low-grade serous carcinomas and Type II – high-grade serous carcinomas) [28], and recurrence and timing of recurrence. Tumour recurrence was defined as either clinical or radiologic evidence of tumor regrowth, or death from any cause [4], within 3 years following the completion of primary treatment. Obesity was defined as having a BMI of 30 kg/m² or higher [29].

### Study outcomes

The study endpoints were progression-free survival (PFS) defined as the period from the completion of primary treatment to the initial indication of disease progression, evidenced by either clinical or radiological detection of tumour regrowth with or without elevated serum CA125 levels; and overall survival (OS), defined as the period between the completion of primary treatment to death from any cause or the last follow-up. We censored the survival data collection after the third year of follow-up [30]. This 3-year follow-up duration falls within the scheduled period recommended for EOC patients’ surveillance in our setting comprising three monthly visits for the first 2 years, then six-monthly visits for the next 3 years, and then annually for low-risk patients. High-risk patients or those with significant comorbidities may require more frequent follow-up visits [26].

### Statistical analyses

Descriptive statistics were computed for patients’ relevant socio-demographic and clinical data. For continuous variables, we used the mean and SD for normally distributed data, or the median and interquartile range for skewed data. Counts and percentages were used to report categorical variables. We used Kaplan-Meier analysis stratified by patients’ BMI categories (obese versus non-obese) and compared using the Log Rank test to estimate PFS and OS [31]. Bivariable and multivariable Cox proportional hazard models were used to estimate hazard ratios (HR) of the associations between the BMI categories and survival outcomes while adjusting for all confounding clinicopathologic variables. Using the backward stepwise conditional techniques, variables with *p* < 0.10 were built into the final multivariable model while a two-tailed hypothesis testing was performed at a 5% alpha level. All statistical analyses were conducted using SPSS version 29.0 for Windows (IBM Corp., Armonk, NY, USA).

### Ethical considerations

Before access to the patient’s medical records and subsequent data collection, the study reported in this article was approved by the Health Research Ethics Committee of the LUTH with approval number ADM/DCST/HREC/APP/3699. The study was conducted per the Declaration of Helsinki. Strict confidentiality of patients' information was ensured during and after the completion of the study.

## Results

**Figure 1.**
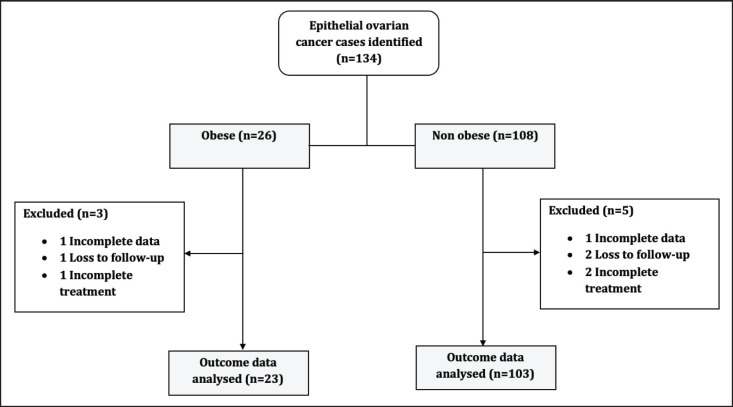
Study patients' flow chart.

We retrieved the data of *n* = 134 women diagnosed with advanced EOC who had 3 years of follow-up after completing the standard first-line treatment. Of these women, *n* = 26 were obese while women *n* = 108 were non-obese (Figure 1).

The patients’ baseline characteristics are presented in Table 1. The mean age of the women was 51.1 ± 13.3 years, and the median serum CA-125 level was 467 (246, 1,550) U/mL. Large proportions of the patients were multiparous (62.7%) and pre-menopausal (52.4%). There were statistically significant differences between obese and non-obese women with advanced EOC in terms of mean age (*p* = 0.013), mean serum CA 125 levels (*p* < 0.001), menstrual status (*p* = 0.005) and presence of comorbidities (*p* = 0.003).

**Figure 2.**
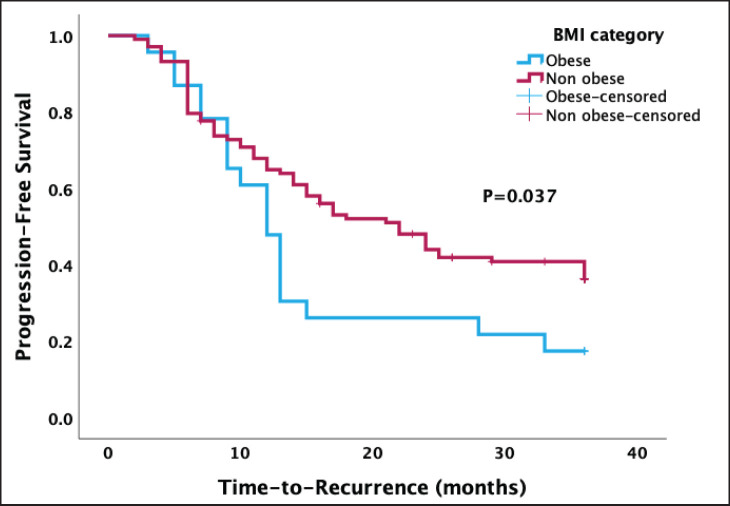
Kaplan-Meier curve of PFS stratified by type of BMI categories. A significant association was found between obesity and PFS (*p* = 0.037).

The Kaplan-Meier survival analysis recorded a median PFS of 17.0 months (95% confidence interval 10.8, 23.2). There was a statistically lower median PFS after 3 years in obese women than in non-obese women (12.0 versus 22.0 months, *p* = 0.037) (Figure 2).

Out of the 126 women in the study, 83 (65.9%) experienced a recurrence within 3 years after completing their primary treatment. Following adjustments for age and parity in the multivariable Cox proportional regression model, there was no statistically significant difference in PFS between obese and non-obese women (adjusted HR = 0.62, 95% confidence interval = 0.36–1.06, *p* = 0.282) (Table 2).

**Figure 3.**
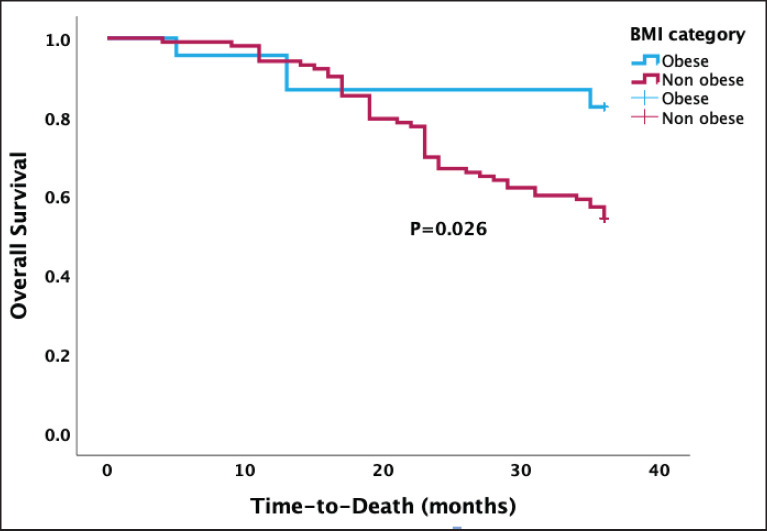
Kaplan-Meier OS curve stratified by BMI categories showing a significant association between obesity and OS (*p* = 0.026).

In Figure 3, the Kaplan-Meier survival analysis revealed a median OS of 30.1 months, with a 95% confidence interval ranging from 28.6 to 31.7 months. There was a statistically higher median OS after 3 years in obese than in non-obese women treatment (30.6 versus 29.6 months, *p* = 0.026).

As shown in Table 3, death was recorded in 51 (40.5%) women during the 3-year follow-up period after treatment. In the multivariable Cox proportional regression model, after adjustments for covariates including menstrual status and presence of ascites, there was a statistically significant difference in the OS between obese and non-obese women (adjusted HR = 3.58, 95% confidence interval 1.28–10.02, *p* = 0.015).

## Discussion

This study investigated the impact of obesity on survival outcomes in women with advanced EOC in Lagos, Nigeria. Our results indicate that while there was no significant association between obesity and PFS, obesity was significantly associated with poorer OS among women with advanced EOC. Our study also showed that obesity with or without significant ascites at diagnosis and treatment appears to be related to poorer OS.

PFS reflects the duration during which a patient’s disease does not worsen, and it can be influenced by various factors including tumour biology, treatment efficacy and patient adherence to therapy [32]. However, our findings suggest that obesity may not directly impact the progression of EOC. This lack of association aligns with some previous studies [33–38], but contradicts the findings of others [39]. These variations in findings may be due to the time point of measurement of BMI and the cutoff points used to define obesity. Some studies used data on height and weight obtained 1 year before diagnosis or from reports of women's usual adult weight [40, 41]. In contrast, others measured BMI at diagnosis [38] and commencement of chemotherapy [34, 35]. We, therefore, propose that the weak relationship between obesity at diagnosis and progression of advanced EOC observed in our study may be due to the use of BMI which may not be an appropriate measure for evaluating the degree of obesity in ovarian cancer patients at the point of diagnosis and treatment as these patients often have ascites or cachexia [42, 43]. Second, this could be due to the lack of any difference in the extent of debulking surgery, the most important predictor of disease progression [33, 44, 45], between obese and non-obese women as observed in our study. Third, our finding may also be due to the 3-year follow-up period which may be too short to observe a significant number of cases with disease progression and death which could prevent us from also capturing the full impact of some historically strong predictors, including, residual disease status and histological subtype [32], on survival outcomes.

The significant association between obesity, in the presence or absence of significant ascites, and poorer OS in our study is consistent with a growing body of literature indicating that obesity adversely affects survival in ovarian cancer patients [39, 40, 46]. This is, however, at variance with the findings from other studies where obesity was not associated with OS [36–38, 41]. Factors that could be attributed to the association observed between obesity and OS in our study and others are – First, due to concerns about potential overdosing in obese patients with a larger body surface area, the dosing of chemotherapeutic drugs for gynecologic malignancies is often capped in some centres [47] including ours based on an empiric body surface area of either 1.8 or 2.0 m² instead of the full dosage calculation based on the body surface area as in non-obese individuals. Second, the associated medical comorbidities seen in obese individuals, such as diabetes, and cardiovascular and metabolic diseases as equally observed in our study, can complicate treatment thus leading to a reduced chemotherapy dose intensity with cumulative doses being lower in obese compared to non-obese women [39, 48]. Obese women might, therefore, be receiving less effective treatment, which could lead to a higher risk of disease progression and reduced survival [49]. Furthermore, obesity is associated with changes in tumour biology, including increased production of oestrogen, insulin resistance and chronic inflammation, all of which can promote tumour growth and metastasis leading to poorer survival outcomes in cancer patients [50].

The strength of our study is that the findings have the potential to inform clinical practice and public health strategies, emphasizing the need for comprehensive management of obese women with EOC. However, we acknowledged a few study limitations. First, the retrospective design may introduce selection and information biases. Second, historical changes in practices, exposures, or population characteristics could account for the unexpectedly high proportions of multiparous and pre-menopausal women seen in the study, and this may limit the applicability of our findings beyond the study contexts. Third, our study could be underpowered due to a lack of sample size calculation thus limiting our ability to translate the statistical to causal inference. Fourth, BMI, as a measure of obesity does not distinguish between muscle and fat mass, nor does it account for fat distribution and ascites especially when measured at diagnosis or commencement of treatment as in our study. Fifth, our study did not account for variations in chemotherapy, which could influence survival outcomes. Finally, the duration of follow-up in our study may not be long enough to capture the full impact of some strong predictors, including, residual disease status and histological subtype [46], on survival outcomes. Future prospective studies with larger sample sizes could enhance statistical power and provide more definitive insights into the relationship between obesity and other prognostic factors and survival outcomes. These studies should also extend the follow-up duration to provide a more comprehensive understanding of long-term survival outcomes related to obesity and should consider using more precise measures of body composition and obesity, and account for the types, courses and adherence to chemotherapy to provide a more comprehensive understanding of the findings of our study.

## Conclusion

This study highlights that obesity is associated with significantly poorer OS in women with advanced EOC in Lagos, Nigeria, despite no observed impact on PFS. These findings underscore the importance of addressing obesity in the management of EOC by introducing comprehensive, multidisciplinary approaches incorporating weight management and personalized treatment strategies to enhance the prognosis of obese women with EOC. However, future prospective studies with larger sample sizes with extended follow-up duration could enhance statistical power and provide more definitive insights into the relationship between obesity and other prognostic factors and survival outcomes. These studies should also consider using more precise measures of body obesity and also account for the types, courses and adherence to chemotherapy to provide a more comprehensive understanding of the findings of our study.

## Conflicts of interest

The authors declare no conflicts of interest.

## Data availability

The datasets used and/or analyzed during the current study are available from the corresponding author (KSO) upon reasonable request.

## Figures and Tables

**Table 1.** Patients baseline characteristics (*n* = 126).

Characteristics	BMI category		*p*-value
	Obese (%)	Non-obese (%)	
	23 (18.3)	103 (81.7)	
Mean age (± SD) in years	57.3 ± 10.9	49.8 ± 13.4	0.013
Median CA-125 levels (IQR) in U/mL	1,007 (467, 1,550)	449 (210, 1,452)	<0.001
Parity			
Nulliparity	6 (26.1%)	41 (39.8%)	0.219
Multiparity	17 (73.9%)	62 (60.2%)	
Menstrual status			
Pre-menopause	6 (26.1%)	60 (58.3%)	0.005
Post-menopause	17 (73.9%)	43 (41.7%)	
Medical comorbidity			
Yes	11 (47.8%)	19 (18.4%)	0.003
No	12 (52.2%)	84 (81.6%)	
Ascites			
Yes	17 (73.9%)	54 (52.4%)	0.060
No	6 (26.1%)	49 (47.6%)	
FIGO stage			
Stage 3	13 (56.5%)	72 (69.9%)	0.216
Stage 4	10 (43.5%)	31 (30.1%)	
Upfront treatment			
PDS	9 (39.1%)	50 (48.5%)	0.413
IDS	14 (60.9%)	53 (51.5%)	
Debulking surgery status			
Optimal	4 (17.4%)	34 (33.0%)	0.140
Suboptimal	19 (82.6%)	69 (67.0%)	
Histological subtype			
Type I (LGSC and others)	8 (34.8%)	38 (36.9%)	0.849
Type II (HGSC)	15 (65.2%)	65 (63.1%)	

BMI (body mass index), CA (cancer antigen), FIGO (International Federation of Gynaecology and Obstetrics), HGSC (high-grade serous carcinomas), IDS (interval debulking surgery), IQR (interquartile range), LGSC (low-grade serous carcinomas), PDS (primary debulking surgery) and SD (standard deviation). Type I includes endometrioid carcinoma, clear cell carcinoma, mucinous carcinoma and low-grade serous carcinomas. Type II includes high-grade serous carcinomas.

**Table 2.** Univariable and multivariable analyses of BMI categories and PFS.

Factors	Number of women with recurrence within 3 years	Crude	Adjusted	
		*p*-value	HR (95% CI)	*p*-value
BMI category				
Obese (≥30 kg/m^2^)	19/23 (82.6%)	0.044	0.69 (0.40–1.16)	0.160
Non-obese (<30 kg/m^2^)	64/103 (62.1%)		1.00 (reference)	
Age				
<52 years	34/62 (54.8%)	0.005	1.88 (1.21–2.92)	0.005
≥52 years	49/64 (76.6%)		1.00 (reference)	
Parity				
Nulliparity	25/47 (53.2%)	0.015	1.50 (0.91–2.47)	0.112
Multiparity	58/79 (73.4%)		1.00 (reference)	
Menstrual status				
Pre-menopause	41/66 (62.1%)	0.523	NA	NA
Post-menopause	42/60 (70.0%)			
**Serum CA-125 levels**				
**≥470 U/mL**	39/61 (63.9%)	0.588	NA	NA
**<470 U/mL**	44/65 (67.7%)			
**Medical comorbidity**				
**Yes**	23/30 (76.7%)	0.262	NA	NA
**No**	60/96 (62.5%)			
**Presence of ascites**				
**Yes**	48/71 (67.6%)	0.263	NA	NA
**No**	35/55 (63.6%)			
**FIGO stage**				
**Stage 3**	54/85 (63.5%)	0.313	NA	NA
**Stage 4**	29/41 (70.7%)			
**Surgical debulking status**				
**Optimal**	28/38 (73.7%)	0.175	NA	NA
**Suboptimal**	55/88 (62.5%)			
**Upfront surgery**				
**PDS**	46/67 (68.7%)	0.396	NA	NA
**IDS**	37/59 (62.7%)			
**Histological subtype**				
**Type I**	27/46 (58.7%)	0.305	NA	NA
**Type II**	56/80 (70.0%)			

BMI (body mass index), CA (cancer antigen), CI (confidence interval), FIGO (International Federation of Gynaecology and Obstetrics), HR (hazard ratio), NA (not applicable), PDS (primary debulking surgery); Type I includes low-grade serous carcinomas and other similar cancers; Type II includes high-grade serous carcinomas.

**Table 3.** Univariable and multivariable analyses of BMI categories and OS.

Factors	Number of women that died within 3 years	Crude	Adjusted	
		*p*-value	HR (95% CI)	*p*-value
BMI				
Obese (≥30 kg/m^2^)	4/23 (17.4%)	0.036	3.58 (1.28–10.02)	0.015
Non-obese (<30 kg/m^2^)	47/103 (45.6%)		1.00 (reference)	
Age				
<52 years	26/62 (41.9%)	0.884	NA	NA
≥52 years	25/64 (39.1%)			
Parity				
Nulliparity	17/47 (36.2%)	0.272	NA	NA
Multiparity	34/79 (43.0%)			
Menstrual status				
Pre-menopause	33/66 (50.0%)	0.032	0.65 (0.36–1.18)	0.156
Post-menopause	18/60 (30.0%)		1.00 (reference)	
**Serum CA-125 levels**				
**≥470 U/mL**	21/61 (34.4%)	0.254	NA	NA
**<470 U/mL**	30/65 (46.2%)			
**Pre-existing morbidity**				
**Yes**	10/30 (33.3%)	0.672	NA	NA
**No**	41/96 (42.7%)			
Presence of ascites				
**Yes**	34/71 (47.9%)	0.055	0.49 (0.27–0.87)	0.016
**No**	17/55 (30.9%)		1.00 (reference)	
**FIGO stage**				
**Stage 3**	30/85 (35.3%)	0.117	NA	NA
**Stage 4**	21/41 (51.2%)			
**Upfront surgery**				
**PDS**	29/67 (43.3%)	0.622	NA	NA
**IDS**	22/59 (37.3%)			
**Surgical debulking status**				
**Optimal**	15/38 (39.5%)	0.943	NA	NA
**Sub-optimal**	36/88 (40.9%)			
**Histological subtype**				
**Type I**	20/46 (43.5%)	0.798	NA	NA
**Type II**	31/80 (38.8%)			

BMI, body mass index; CA, cancer antigen; CI, confidence interval; FIGO, International Federation of Gynecology and Obstetrics; HR, hazard ratio; NA, not applicable; PDS; primary debulking surgery; Type I includes low-grade serous carcinomas and others; Type II includes high-grade serous carcinomas
